# Diaqua­bromido­copper(II)–18-crown-6–water (1/1/2)

**DOI:** 10.1107/S1600536810023500

**Published:** 2010-06-23

**Authors:** Bo Wang

**Affiliations:** a Ordered Matter Science Research Center, Southeast University, Nanjing 211189, People’s Republic of China

## Abstract

In the title compound, [CuBr_2_(H_2_O)_2_]·C_12_H_24_O_6_·2H_2_O, the Cu^II^ atom, which is situated on an inversion centre and has a slightly distorted square-planar geometry, and the two coordinated water mol­ecules are linked to the 18-crown-6 macrocycles by O—H⋯O hydrogen bonds. The water mol­ecule of crystallization further links the metal complex and the crown ether macrocycles into a chain along the *c* axis.

## Related literature

For the ability of 18-crown-6 ether to form complexes with different metal ions, see: Jackson *et al.* (1981 ▶); Otter & Hartshorn (2004 ▶). For similar structures, see: Antsyshkina *et al.* (2004 ▶); Liu *et al.* (2007 ▶). For bond-length data, see: Allen *et al.* (1987 ▶).
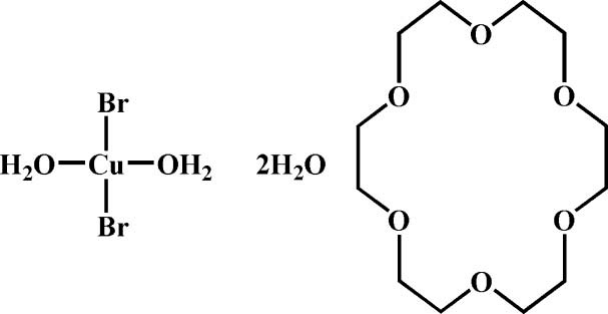


## Experimental

### 

#### Crystal data

[CuBr_2_(H_2_O)_2_]·C_12_H_24_O_6_·2H_2_O
*M* *_r_* = 559.73
Triclinic, 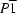

*a* = 7.4418 (5) Å
*b* = 8.1724 (6) Å
*c* = 10.1510 (2) Å
α = 75.220 (3)°
β = 69.47 (1)°
γ = 78.51 (1)°
*V* = 554.90 (6) Å^3^
*Z* = 1
Mo *K*α radiation
μ = 4.63 mm^−1^
*T* = 298 K
0.20 × 0.20 × 0.20 mm

#### Data collection

Rigaku SCXmini diffractometer
Absorption correction: multi-scan *CrystalClear* (Rigaku, 2005 ▶) *T*_min_ = 0.397, *T*_max_ = 0.412
5746 measured reflections
2537 independent reflections
2064 reflections with *I* > 2σ(*I*)
*R*_int_ = 0.037

#### Refinement

*R*[*F* ^2^ > 2σ(*F* ^2^)] = 0.052
*wR*(*F* ^2^) = 0.155
*S* = 1.08
2537 reflections
123 parameters
H atoms treated by a mixture of independent and constrained refinement
Δρ_max_ = 1.27 e Å^−3^
Δρ_min_ = −1.55 e Å^−3^

### 

Data collection: *CrystalClear* (Rigaku 2005 ▶); cell refinement: *CrystalClear*; data reduction: *CrystalClear*; program(s) used to solve structure: *SHELXS97* (Sheldrick, 2008 ▶); program(s) used to refine structure: *SHELXL97* (Sheldrick, 2008 ▶); molecular graphics: *SHELXTL* (Sheldrick, 2008 ▶); software used to prepare material for publication: *PRPKAPPA* (Ferguson, 1999 ▶).

## Supplementary Material

Crystal structure: contains datablocks I, New_Global_Publ_Block. DOI: 10.1107/S1600536810023500/jj2036sup1.cif

Structure factors: contains datablocks I. DOI: 10.1107/S1600536810023500/jj2036Isup2.hkl

Additional supplementary materials:  crystallographic information; 3D view; checkCIF report

## Figures and Tables

**Table 1:** Hydrogen-bond geometry (Å, °)

*D*—H⋯*A*	*D*—H	H⋯*A*	*D*⋯*A*	*D*—H⋯*A*
O4*W*—H4*WA*⋯O1^i^	0.68 (7)	2.30 (8)	2.962 (6)	167 (9)
O4*W*—H4*WB*⋯O3^i^	0.93 (8)	1.95 (8)	2.869 (6)	170 (6)
O5*W*—H5*WA*⋯O2	0.85	1.92	2.715 (5)	156
O5*W*—H5*WB*⋯O4*W*	0.85	1.82	2.609 (6)	155

Symmetry code: (i) 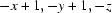.

**Fig. 1.**
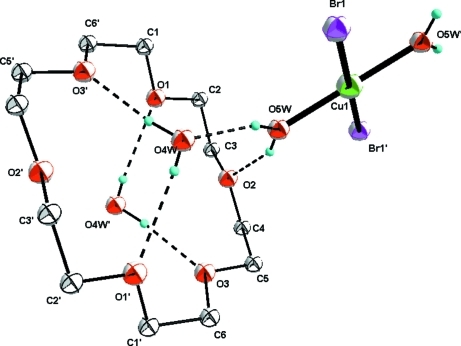
The molecular structure of the title compound, with the atomic numbering scheme. Displacement ellipsoids are drawn at the 30% probability level, and the H atoms attached to C have been omitted for clarity. Dashed lines indicate O—H···O hydrogen bonds.

**Fig. 2.**
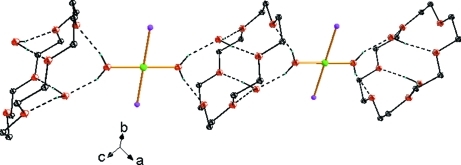
One-dimensional chain of (I) showing the Owater—H···Ocrown hydrogen bonds (dashed lines). H atoms attached to C have been omitted.

## supplementary crystallographic information

### Comment

The ability of 18-crown-6 ether (18-C-6) to form complexes with different metal ions has been widely investigated (Jackson *et al.*, 1981; Otter & Hartshorn, 2004). We report here the synthesis and crystal structure of an intermediate in the 18-crown-6 ether-mediated solubilization of copper bromide salts, namely [CuBr_2_(H_2_O)_2 _].(18-crown-6).2H_2_O, (I).

The crystal structure of (I) consists of CuBr_2_(H_2_O)_2_ complex, one molecule of 18-crown-6 ether and two water molecule in the crystallographic asymmetric unit (Fig. 1). The structure is similar to that found in [CuCl_2_(H_2_O)_2_].(18-crown-6).2H_2_O (Antsyshkina *et al.*, 2004; Liu *et al.*, 2007), where the Cu atom is bonded to two Br and two H_2_O molecules in a square planar coordination. The mean Cu—Br and Cu—O bond lengths are 2.3687 (5) Åand 1.911 (4) Å, respectively. Bond length and angles are in normal ranges (Allen *et al.*, 1987). All O atoms in the crown form O—H···O_crown_ hydrogen bonds with adjacent coordinated (O5W) and uncoordinated (O4W) water molecules, with average O···O distances of 2.609 Å. Thus, the hydrogen bonds link the crown ethers and Cu complex into a one-dimensional chain along the *c* axis (Fig. 2).

### Experimental

CuBr_2_(44.6 mg, 0.2 mmol) and 18-crown-6 (53 mg, 0.2 mmol) were added to 10 ml of THF and 2 ml H_2_O, and this reaction mixture was stirred at 333 K for 6 h. After filtration, the resulting filtrate was reduced to 5 ml in a small tube, which was loaded into a large vial containing 5 ml of diethyl ether. The large vial was sealed and left undisturbed at room temperature, and colorless crystals of (I) were obtained in 5 d.

### Refinement

Water H atoms were located in a difference Fourier map and refined as riding in their as-found relative positions, with *U*_iso_(H) = 1.2*U*_eq_(O). Other H atoms were placed in calculated positions, with C—H = 0.97 Å, and refined in riding mode, with *U*_iso_(H) = 1.2*U*_eq_(C).

### Crystal data

**Table:**

[CuBr_2_(H_2_O)_2_]·C_12_H_24_O_6_·2H_2_O	*Z* = 1
*M**_r_* = 559.73	*F*(000) = 283
Triclinic, *P*1	*D*_x_ = 1.675 Mg m^−^^3^
Hall symbol: -P 1	Mo *K*α radiation, λ = 0.71073 Å
*a* = 7.4418 (5) Å	Cell parameters from 2622 reflections
*b* = 8.1724 (6) Å	θ = 3.0–27.5°
*c* = 10.1510 (2) Å	µ = 4.63 mm^−^^1^
α = 75.220 (3)°	*T* = 298 K
β = 69.47 (1)°	Prism, green
γ = 78.51 (1)°	0.20 × 0.20 × 0.20 mm
*V* = 554.90 (6) Å^3^	

### Data collection

**Table:**

Rigaku SCXmini diffractometer	2537 independent reflections
Radiation source: fine-focus sealed tube	2064 reflections with *I* > 2σ(*I*)
graphite	*R*_int_ = 0.037
Detector resolution: 13.6612 pixels mm^-1^	θ_max_ = 27.5°, θ_min_ = 3.0°
ω scans	*h* = −9→9
Absorption correction: multi-scan *CrystalClear* (Rigaku, 2005)	*k* = −10→10
*T*_min_ = 0.397, *T*_max_ = 0.412	*l* = −13→13
5746 measured reflections	

### Refinement

**Table:**

Refinement on *F*^2^	Primary atom site location: structure-invariant direct methods
Least-squares matrix: full	Secondary atom site location: difference Fourier map
*R*[*F*^2^ > 2σ(*F*^2^)] = 0.052	Hydrogen site location: inferred from neighbouring sites
*wR*(*F*^2^) = 0.155	H atoms treated by a mixture of independent and constrained refinement
*S* = 1.08	*w* = 1/[σ^2^(*F*_o_^2^) + (0.0755*P*)^2^ + 0.8701*P*] where *P* = (*F*_o_^2^ + 2*F*_c_^2^)/3
2537 reflections	(Δ/σ)_max_ < 0.001
123 parameters	Δρ_max_ = 1.27 e Å^−^^3^
0 restraints	Δρ_min_ = −1.55 e Å^−^^3^

### Special details

**Table:**

Geometry. All e.s.d.'s (except the e.s.d. in the dihedral angle between two l.s. planes) are estimated using the full covariance matrix. The cell e.s.d.'s are taken into account individually in the estimation of e.s.d.'s in distances, angles and torsion angles; correlations between e.s.d.'s in cell parameters are only used when they are defined by crystal symmetry. An approximate (isotropic) treatment of cell e.s.d.'s is used for estimating e.s.d.'s involving l.s. planes.
Refinement. Refinement of *F*^2^ against ALL reflections. The weighted *R*-factor *wR* and goodness of fit *S* are based on *F*^2^, conventional *R*-factors *R* are based on *F*, with *F* set to zero for negative *F*^2^. The threshold expression of *F*^2^ > σ(*F*^2^) is used only for calculating *R*-factors(gt) *etc*. and is not relevant to the choice of reflections for refinement. *R*-factors based on *F*^2^ are statistically about twice as large as those based on *F*, and *R*- factors based on ALL data will be even larger.

### Fractional atomic coordinates and isotropic or equivalent isotropic displacement parameters (Å^2^)

**Table:**

	*x*	*y*	*z*	*U*_iso_*/*U*_eq_	
Br1	0.05334 (10)	0.20118 (6)	0.58028 (6)	0.0652 (3)	
C1	0.7159 (11)	0.5878 (10)	0.2213 (9)	0.080 (2)	
H1A	0.7893	0.5678	0.2875	0.096*	
H1B	0.5881	0.6430	0.2652	0.096*	
C2	0.6378 (11)	0.3049 (11)	0.3232 (7)	0.082 (2)	
H2A	0.5237	0.3535	0.3903	0.099*	
H2B	0.7387	0.2700	0.3681	0.099*	
C3	0.5931 (11)	0.1552 (9)	0.2897 (7)	0.078 (2)	
H3A	0.7043	0.1104	0.2180	0.094*	
H3B	0.5624	0.0664	0.3755	0.094*	
C4	0.3741 (11)	0.0667 (7)	0.2063 (8)	0.077 (2)	
H4A	0.3196	−0.0119	0.2951	0.092*	
H4B	0.4851	0.0053	0.1465	0.092*	
C5	0.2298 (10)	0.1320 (9)	0.1320 (9)	0.079 (2)	
H5A	0.1834	0.0378	0.1166	0.095*	
H5B	0.1206	0.1968	0.1901	0.095*	
C6	0.1858 (10)	0.3000 (10)	−0.0843 (10)	0.083 (2)	
H6A	0.0709	0.3637	−0.0304	0.100*	
H6B	0.1466	0.2052	−0.1045	0.100*	
Cu1	0.0000	0.5000	0.5000	0.0335 (2)	
O1	0.7004 (6)	0.4308 (6)	0.1941 (4)	0.0626 (10)	
O2	0.4337 (5)	0.2051 (5)	0.2370 (4)	0.0559 (9)	
O3	0.3148 (5)	0.2374 (5)	−0.0014 (5)	0.0613 (10)	
O4W	0.3072 (6)	0.6708 (6)	0.0637 (4)	0.0566 (10)	
O5W	0.1698 (7)	0.4741 (5)	0.3133 (4)	0.0900 (18)	
H5WA	0.2229	0.3756	0.2979	0.108*	
H5WB	0.1920	0.5610	0.2455	0.108*	
H4WA	0.309 (11)	0.633 (10)	0.011 (8)	0.07 (3)*	
H4WB	0.436 (11)	0.688 (9)	0.040 (8)	0.08 (2)*	

### Atomic displacement parameters (Å^2^)

**Table:**

	*U*^11^	*U*^22^	*U*^33^	*U*^12^	*U*^13^	*U*^23^
Br1	0.0949 (5)	0.0358 (3)	0.0446 (3)	−0.0137 (3)	0.0039 (3)	−0.0051 (2)
C1	0.085 (5)	0.091 (5)	0.092 (5)	0.007 (4)	−0.054 (4)	−0.043 (4)
C2	0.088 (5)	0.112 (6)	0.044 (3)	0.010 (4)	−0.035 (3)	−0.007 (4)
C3	0.088 (5)	0.066 (4)	0.053 (4)	0.011 (4)	−0.016 (3)	0.011 (3)
C4	0.093 (5)	0.037 (3)	0.066 (4)	−0.015 (3)	0.016 (4)	−0.006 (3)
C5	0.066 (4)	0.059 (4)	0.095 (5)	−0.033 (3)	0.015 (4)	−0.025 (4)
C6	0.066 (4)	0.084 (5)	0.132 (7)	−0.008 (4)	−0.045 (5)	−0.057 (5)
Cu1	0.0319 (4)	0.0385 (4)	0.0250 (4)	−0.0049 (3)	−0.0047 (3)	−0.0033 (3)
O1	0.068 (2)	0.077 (3)	0.048 (2)	0.000 (2)	−0.023 (2)	−0.022 (2)
O2	0.056 (2)	0.0428 (19)	0.047 (2)	0.0025 (16)	0.0013 (17)	−0.0033 (16)
O3	0.049 (2)	0.060 (2)	0.073 (3)	−0.0165 (18)	−0.0070 (19)	−0.021 (2)
O4W	0.054 (2)	0.074 (3)	0.034 (2)	−0.016 (2)	−0.0026 (17)	−0.0073 (19)
O5W	0.109 (4)	0.054 (2)	0.040 (2)	0.029 (2)	0.026 (2)	0.0074 (18)

### Geometric parameters (Å, °)

**Table:**

Br1—Cu1	2.3687 (5)	C4—H4B	0.9700
C1—O1	1.414 (8)	C5—O3	1.413 (8)
C1—C6^i^	1.488 (11)	C5—H5A	0.9700
C1—H1A	0.9700	C5—H5B	0.9700
C1—H1B	0.9700	C6—O3	1.429 (8)
C2—O1	1.438 (8)	C6—C1^i^	1.488 (11)
C2—C3	1.476 (11)	C6—H6A	0.9700
C2—H2A	0.9700	C6—H6B	0.9700
C2—H2B	0.9700	Cu1—O5W^ii^	1.911 (4)
C3—O2	1.414 (8)	Cu1—O5W	1.911 (4)
C3—H3A	0.9700	Cu1—Br1^ii^	2.3687 (5)
C3—H3B	0.9700	O4W—H4WA	0.68 (7)
C4—O2	1.432 (8)	O4W—H4WB	0.93 (8)
C4—C5	1.463 (11)	O5W—H5WA	0.8500
C4—H4A	0.9700	O5W—H5WB	0.8500
			
O1—C1—C6^i^	109.7 (6)	O3—C5—H5A	109.9
O1—C1—H1A	109.7	C4—C5—H5A	109.9
C6^i^—C1—H1A	109.7	O3—C5—H5B	109.9
O1—C1—H1B	109.7	C4—C5—H5B	109.9
C6^i^—C1—H1B	109.7	H5A—C5—H5B	108.3
H1A—C1—H1B	108.2	O3—C6—C1^i^	109.5 (5)
O1—C2—C3	110.2 (5)	O3—C6—H6A	109.8
O1—C2—H2A	109.6	C1^i^—C6—H6A	109.8
C3—C2—H2A	109.6	O3—C6—H6B	109.8
O1—C2—H2B	109.6	C1^i^—C6—H6B	109.8
C3—C2—H2B	109.6	H6A—C6—H6B	108.2
H2A—C2—H2B	108.1	O5W^ii^—Cu1—O5W	180.000 (1)
O2—C3—C2	108.9 (5)	O5W^ii^—Cu1—Br1^ii^	89.03 (12)
O2—C3—H3A	109.9	O5W—Cu1—Br1^ii^	90.97 (12)
C2—C3—H3A	109.9	O5W^ii^—Cu1—Br1	90.97 (12)
O2—C3—H3B	109.9	O5W—Cu1—Br1	89.03 (12)
C2—C3—H3B	109.9	Br1^ii^—Cu1—Br1	180.0
H3A—C3—H3B	108.3	C1—O1—C2	112.9 (6)
O2—C4—C5	109.9 (5)	C3—O2—C4	113.2 (5)
O2—C4—H4A	109.7	C5—O3—C6	112.7 (5)
C5—C4—H4A	109.7	H4WA—O4W—H4WB	103 (8)
O2—C4—H4B	109.7	Cu1—O5W—H5WA	120.0
C5—C4—H4B	109.7	Cu1—O5W—H5WB	120.0
H4A—C4—H4B	108.2	H5WA—O5W—H5WB	120.0
O3—C5—C4	109.1 (5)		
			
O1—C2—C3—O2	−64.8 (7)	C2—C3—O2—C4	−177.8 (5)
O2—C4—C5—O3	63.1 (7)	C5—C4—O2—C3	−171.2 (5)
C6^i^—C1—O1—C2	169.8 (6)	C4—C5—O3—C6	177.1 (5)
C3—C2—O1—C1	170.5 (6)	C1^i^—C6—O3—C5	178.9 (5)

Symmetry codes: (i) −*x*+1, −*y*+1, −*z*; (ii) −*x*, −*y*+1, −*z*+1.

### Hydrogen-bond geometry (Å, °)

**Table:**

*D*—H···*A*	*D*—H	H···*A*	*D*···*A*	*D*—H···*A*
O4W—H4WA···O1^i^	0.68 (7)	2.30 (8)	2.962 (6)	167 (9)
O4W—H4WB···O3^i^	0.93 (8)	1.95 (8)	2.869 (6)	170 (6)
O5W—H5WA···O2	0.85	1.92	2.715 (5)	156
O5W—H5WB···O4W	0.85	1.82	2.609 (6)	155

Symmetry codes: (i) −*x*+1, −*y*+1, −*z*.

## References

[bb1] Allen, F. H., Kennard, O., Watson, D. G., Brammer, L., Orpen, A. G. & Taylor, R. (1987). *J. Chem. Soc. Perkin Trans. 2*, pp. S1–19.

[bb2] Antsyshkina, A. S., Sadikov, G. G., Koksharova, T. V. & Sergienko, V. S. (2004). *Zh. Neorg. Khim.* **49**, 1797–1800.

[bb3] Ferguson, G. (1999). *PRPKAPPA* University of Guelph, Canada.

[bb4] Jackson, W. G., Sargeson, A. M., Tucker, P. A. & Watson, A. D. (1981). *J. Am. Chem. Soc.* **103**, 533–540.

[bb5] Liu, X., Guo, G.-C. & Sun, Y.-Y. (2007). *Acta Cryst.* E**63**, m275–m277.

[bb6] Otter, C. A. & Hartshorn, R. M. (2004). *J. Chem. Soc. Dalton Trans.* pp. 150–156. 10.1039/b311809f 15356754

[bb7] Rigaku (2005). *CrystalClear* Rigaku Corporation, Tokyo, Japan.

[bb8] Sheldrick, G. M. (2008). *Acta Cryst.* A**64**, 112–122. 10.1107/S0108767307043930 18156677

